# Changes in the Facial Skeleton With Aging: Implications and Clinical Applications in Facial Rejuvenation

**DOI:** 10.1007/s00266-012-9904-3

**Published:** 2012-05-12

**Authors:** Bryan Mendelson, Chin-Ho Wong

**Affiliations:** 1 The Centre for Facial Plastic Surgery, 109 Mathoura Road, Toorak, VIC 3142 Australia; 2 W Aesthetic Plastic Surgery, Mount Elizabeth Novena Hospital, 38 Irrawaddy Road, #08 – 42, Singapore, 329563 Singapore

**Keywords:** Aging, Changes, Correction, Facial, Rejuvenation, Skeleton

## Abstract

**Abstract:**

In principle, to achieve the most natural and harmonious rejuvenation of the face, all changes that result from the aging process should be corrected. Traditionally, soft tissue lifting and redraping have constituted the cornerstone of most facial rejuvenation procedures. Changes in the facial skeleton that occur with aging and their impact on facial appearance have not been well appreciated. Accordingly, failure to address changes in the skeletal foundation of the face may limit the potential benefit of any rejuvenation procedure. Correction of the skeletal framework is increasingly viewed as the new frontier in facial rejuvenation. It currently is clear that certain areas of the facial skeleton undergo resorption with aging. Areas with a strong predisposition to resorption include the midface skeleton, particularly the maxilla including the pyriform region of the nose, the superomedial and inferolateral aspects of the orbital rim, and the prejowl area of the mandible. These areas resorb in a specific and predictable manner with aging. The resultant deficiencies of the skeletal foundation contribute to the stigmata of the aging face. In patients with a congenitally weak skeletal structure, the skeleton may be the primary cause for the manifestations of premature aging. These areas should be specifically examined in patients undergoing facial rejuvenation and addressed to obtain superior aesthetic results.

**Level of Evidence IV:**

This journal requires that authors assign a level of evidence to each article. For a full description of these Evidence-Based Medicine ratings, please refer to the Table of Contents or the online Instructions to Authors www.springer.com/00266.

**Electronic supplementary material:**

The online version of this article (doi:10.1007/s00266-012-9904-3) contains supplementary material, which is available to authorized users.

The facial skeleton is generally believed to expand continuously throughout life [1–4]. This is reflected in the progressive increase in certain facial anthropometric measurements with age such as the nasion-to-anterior nasal spine and the facial width [5, 6]. The fact that certain areas of the facial skeleton also undergo resorption with aging is not well appreciated or even accepted. For example, it has been thought that maxillary retrusion of the maxilla does not occur with aging in the fully dentate patient [6, 7]. However, contrary to this view, recent evidence clearly demonstrates that aging of the maxilla is primarily one of bone resorption [8–15].

Selective bone resorption in the facial skeleton is not without precedent. Most notably, in the process of differential growth, areas of bone resorption occur adjacent to areas of bone deposition. Differential growth enables the infant skull to assume the proportions of the adult form [16, 17]. This process continues to remodel the fully dentate mature, facial bone and alters the craniofacial morphology into one that is instantly recognizable as an aged skull.

Despite this, the concept that specific areas of the adult facial skeleton are susceptible to resorption remains controversial. Some authors have argued that the skeleton itself undergoes minimal changes with aging and that ongoing aging merely unmasks the underlying skeletal structure [18, 19]. To better define the extent of these bony changes with aging and to consolidate piecemeal information in the literature, a review of the literature was undertaken. The application of this knowledge to selectively correct areas of reduced bone projections in patients with deficiencies either inherent or due to aging is discussed.

## Methods and Materials

A Pubmed search was performed using the keywords “face,” “bony changes,” and “aging.” To ensure the inclusion of all relevant studies, a subsearch then was performed with the keywords “forehead, bone, and aging”; “midface, bone, and aging; “nose, bone, and aging”; and “lower face, bone, and aging.” The inclusion criteria specified studies that directly compared anthropometric facial skeleton measurements between matured adult and elderly dentate patients. The exclusion criteria ruled out pathologic cases, including those with congenital anomalies such as cleft lip and palate, craniofacial syndromes, or a history of craniofacial trauma. The papers were reviewed and the relevant data extracted. For the purpose of analysis and description, the face was divided into four regions: periorbital, midface, perinasal, and mandible.

## Results

Consistent with previous reports, the facial skeleton has a general tendency to enlarge or expand continually with age [1–4]. The vertical height of the facial skeleton, for instance, increases continuously with age unless supervening factors such as tooth loss intervene. Males have more prominent brow ridges, differently shaped orbital rims, a larger pyriform aperture, and a larger jaw than females [20]. In addition, males and females differ in the rate and extent of facial bony changes with aging [21, 22]. Within the limits of these differences, however, the changes noted in the following discussion occur among both sexes.

Selective resorption occurs in specific areas of the adult facial bone. Contrary to conventional beliefs, remodeling of the facial skeleton occurs unabated regardless of the state of the dentition, although the loss of dentition significantly accelerates bony resorption of the maxilla and mandible [23].

### Periorbital Region

**Fig. 1:**
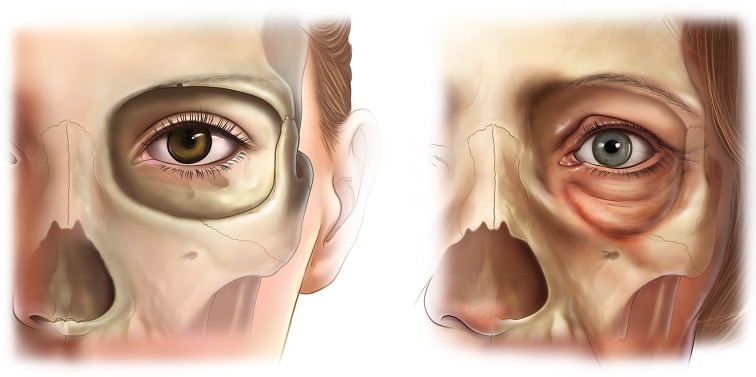
Orbital aging. The superomedial and inferolateral aspects of the orbit have the greatest tendency to resorb. This contributes to the stigmata of periorbital aging such as increased prominence of the medial fat pad, elevation of the medial brow, and lengthening of the lid cheek junction

The orbital aperture increases with age, in both area and width. Resorption is, however, uneven and site specific [8]. The superomedial and inferolateral aspects of the orbital rim, in particular, recede more, although the changes occur at different rates (Fig. 1). The inferolateral orbital rim changes manifest earlier, by middle age, whereas in the superomedial quadrant, recession may be noted only in old age. The inferomedial quadrant of the orbit also has a tendency to recede in old age, especially in males [8, 9]. In contrast, the central part of the superior and inferior orbital rims is more stable, with little if any resorption occurring with age [10, 12].

Pessa [10] found no significant changes in the orbital angle (superior-to-inferior midorbital rim points on the lateral view) with aging, indicating that either the orbital rims do not recede or that one does not recede more rapidly than the other. Mendelson et al. [12] directly measured the lengths of the orbital roof and floor with aging (at the midaxis of each orbit) and found no significant changes in these distances with aging, indicating that the central portion of the superior and inferior orbital rims do not recede with aging.

### Midface

The midface skeleton is formed by the maxilla in the medial and middle thirds and by the body and arch of the zygoma in the lateral third. Contrary to conventional orthodontic teaching [4, 5], it has been clearly demonstrated recently that midface retrusion does occur with aging in dentulous patients [10]. The rate of bony resorption in the midface, however, is not uniform. The maxilla is more susceptible to age-related loss than the zygoma [24].

**Fig. 2:**
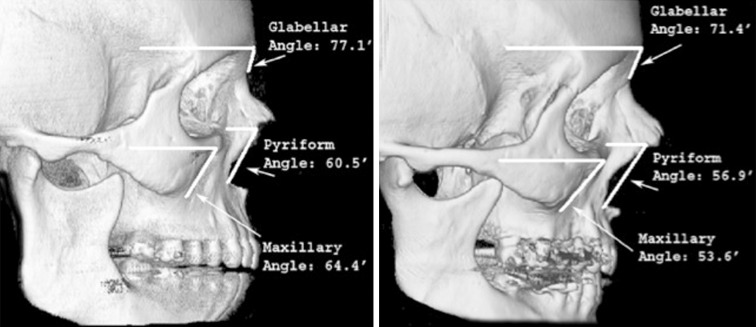
The piriform (piriform angle) and the maxilla (maxillary angle) significantly recede with aging, from youth (*left*) to old age (*right*) [11]

Pessa [10] measured the maxillary angle (superior-to-inferior maxilla at the articulation of the inferior maxillary wing and alveolar arch) of young and old patients and demonstrated significant bone resorption with loss of projection of the maxilla. Shaw and Kahn [11] similarly noted a significant reduction of the maxillary angle with aging (Fig. 2). Using a more precise approach to measurement with standardized parasagittal computed tomography (CT) slices through the midaxis of the orbit to measure the angle between the floor of the orbit and the anterior maxilla, Mendelson et al. [12] confirmed the important finding that the maxilla retrudes with aging and quantitated the changes. The maxillary angle decreased by about 10 ° between young (age < 30 years) and old (age > 60 years) individuals.

### Perinasal Changes

The characteristics of the aging nose are well known and include the following key changes. The nose lengthens and the tip droops, with the collumella and the lateral crurae displacing posteriorly [25]. Changes in the bony foundation that support the nose in youth, the paired nasal bone, and the ascending processes of the maxillae are responsible for many of the soft tissues changes seen in the nose with aging.

Shaw and Khan [11] found that the piriform aperture, resembling the situation of the orbital aperture, enlarges with aging as the edges of the “nasal” bones recede with age. Similarly, bone loss is not uniform, with the greatest resorption occurring in the ascending process of the maxilla. The posterior displacement of the bone rim is greatest at the lower pyriform aperture, which is the critical area for support of the lateral crurae and the external nasal valves [26].

**Fig. 3:**
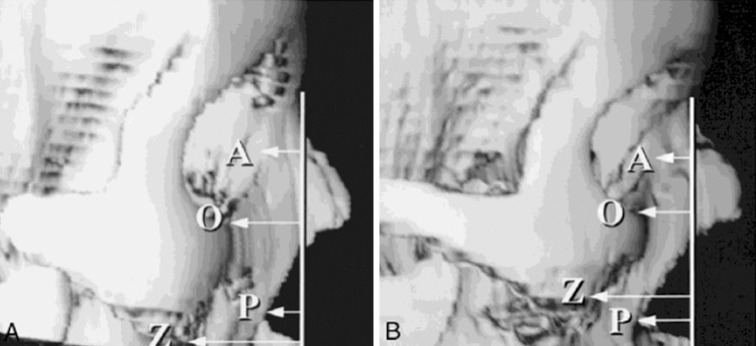
In youth, the piriform (P) lies anterior to the anterior lacrimal crest (A). With aging, the piriform comes to lie posterior to the anterior lacrimal crest as a result of selective bone resorption at the piriform (*below*) [49]

**Fig. 4:**
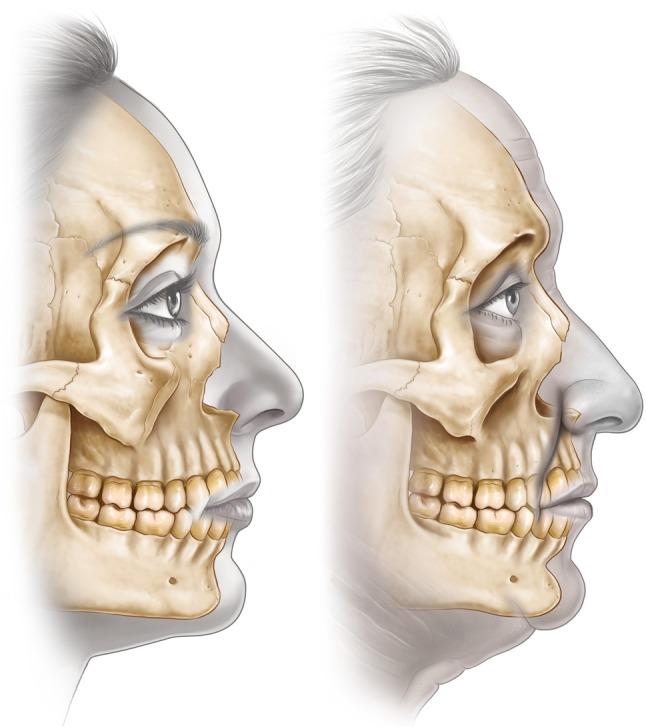
The loss of bone in the pyriform area weakens the support of the lateral crura. Deepening of the maxilla results in posterior positioning of the nasolabial crease and adjacent upper lip

Pessa measured the length of the perpendicular line from the nasion to the pyriform on standardized lateral views of three-dimensional CT images and observed that the distance increases significantly with aging, indicating preferential bone loss in the lower part of the pyriform aperture (Fig. 3). This manifests clinically as posterior displacement of the alar base (relative to the fixed position of the medial canthus) (Fig. 4) [10, 14, 26]. Bone loss here contributes also to deepening of the nasolabial fold with age, which previously had been attributed solely to soft tissue laxity and descent [27]. The anterior nasal spine also recedes with aging (although at a slower rate), and this reduced skeletal support contributes to retraction of the collumella, with downward tip rotation and apparent lengthening of the nose with aging [25, 28].

### Lower Face

The dentate mandible is assumed to expand continuously with aging. This was substantiated by two longitudinal studies. Pecora et al. [29] found that the length of the mandible increases with age for both genders based on lateral cephalograms of 19 male and 20 female subjects. Pessa et al. [15], using frontal radiographs of eight males and eight females from the Bolton Brush Growth Study, found an increase in mandible width and height with increasing age.

The most recent study on aging of the mandible compared three-dimensional CT scans in a population of 120 young, middle-aged, and elderly subjects [30]. In contrast to earlier studies, although certain measurements increased significantly with aging, some measurements contracted. There were no significant changes in the bigonial width and ramus breadth with aging. Whereas the mandibular angle increased, the ramus height and mandibular body height and length decreased.

These findings contradict the earlier studies, which suggest that the mandible expands continuously with age. This may be related to the measurement of normal growth in young subjects who have not yet reached skeletal maturity [15, 29], inadvertently giving a result that the mandible is larger than in the old-age group. For example, in the longitudinal study of Pessa et al. [15], the age range of the female young group was 5–17 years. Comparing these young subjects with mature subjects would most likely result in the latter appearing larger, thereby giving the impression of continuous expansion with aging. Shaw et al. [30] compared subject groups in three age ranges: 20–40, 41–64 years, and older than 64 years (i.e., all subjects had attained full maturity).

These standard parameters, based on linear measurements, will fail to detect in-between areas of reduced skeletal projection such as the prejowl region of the mandible that develops into an area of relative concavity [15] and contributes to the appearance of jowls [13]. Jowls appear at a younger age in patients with microgenia because of the relatively inadequate skeletal support in this area [31].

## Discussion

More than 40 years ago, Enlow [16] followed the growth of the facial skeleton longitudinally from infancy to young adulthood by means of serial cephalometrograms and noted that the entire face becomes longer vertically, deeper in the anterior posterior plane, and wider in the transverse dimension. The following specific changes occur with growth: increasing protrusion of the glabella; expansion of the supraorbital ridges; lateral translation of the orbits; increase in the depth and lateral expansion of the cheeks; increase in length, width, and vertical dimensions of the nose; and increase in vertical height in the occlusal region associated with increased chin prominence. Enlow’s [16] findings formed the basis for the widely accepted teaching that craniofacial growth is one of continuous expansion throughout life.

**Fig. 5:**
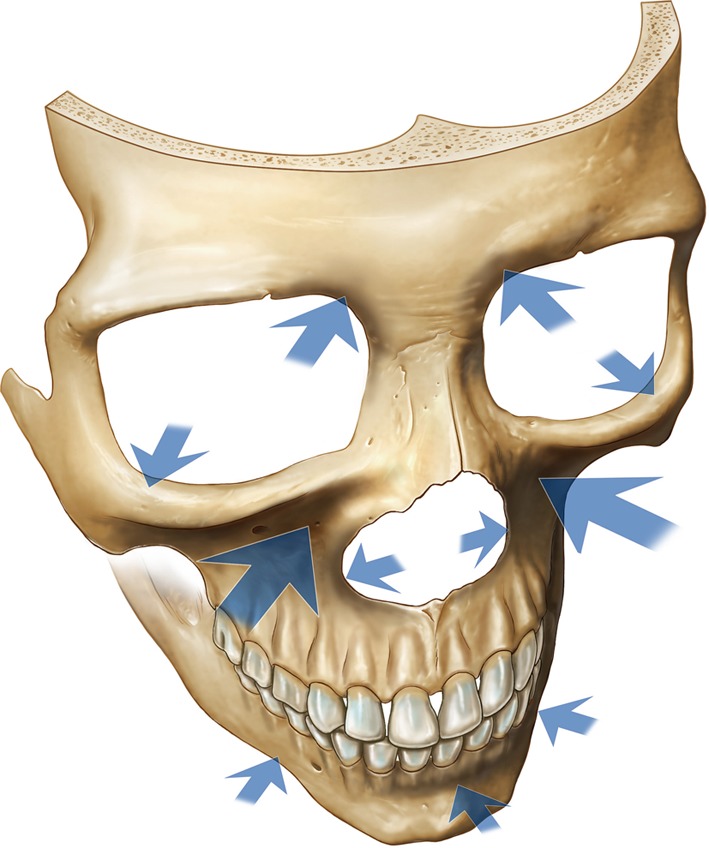
*Arrows* indicate the areas of the facial skeleton susceptible to resorption with aging. The *size of the arrow* correlates with the amount of resorption

The traditional concepts of facial aging revolve around the theme of changes occurring in the soft tissues, with atrophic laxity leading to tissue descent. Facial rejuvenation techniques have focused on reversing these changes by repositioning and redraping of the tissues, with an emphasis on vectors of lift. Although these approaches are effective to a major degree, they do not necessarily produce a completely harmonious or natural rejuvenation. The recent addition of volume to the soft tissues using lipid filling has enabled better restoration of youthful volume and shape than mere lifting alone. Only during the current decade, with the application of three-dimensional CT analyses, has a more accurate understanding of facial skeletal aging been possible. The current review has gathered the isolated pieces of evidence which together unequivocally show that aging of the facial skeleton includes selective resorption at specific sites (Fig. 5) (see supplemental animation video). These changes are mainly in the periorbital and mid cheek and specifically include the superomedial and inferolateral aspects of the orbit, the medial suborbital and pyriform areas of the maxilla, and the prejowl area of the mandible [8–15]. These sites presumably reach their peak “projection” in early adulthood and gradually lose volume thereafter. Rebuilding these areas of lost skeletal support is another method for restoring projection and facilitating repositioning of the soft tissue. This method also is a fundamental element in achieving the goals of natural-appearing facial rejuvenations [32–35].

What are the general consequences of the resorption of the facial skeleton? The periosteum retrudes, altering the position of the outer surfaces of the bones. Accordingly, the location of the attachments of facial ligaments and muscles through the periosteum also moves. As a result, these structures may lose the mechanical advantage of their effect on the tissues they act upon.

**Fig. 6:**
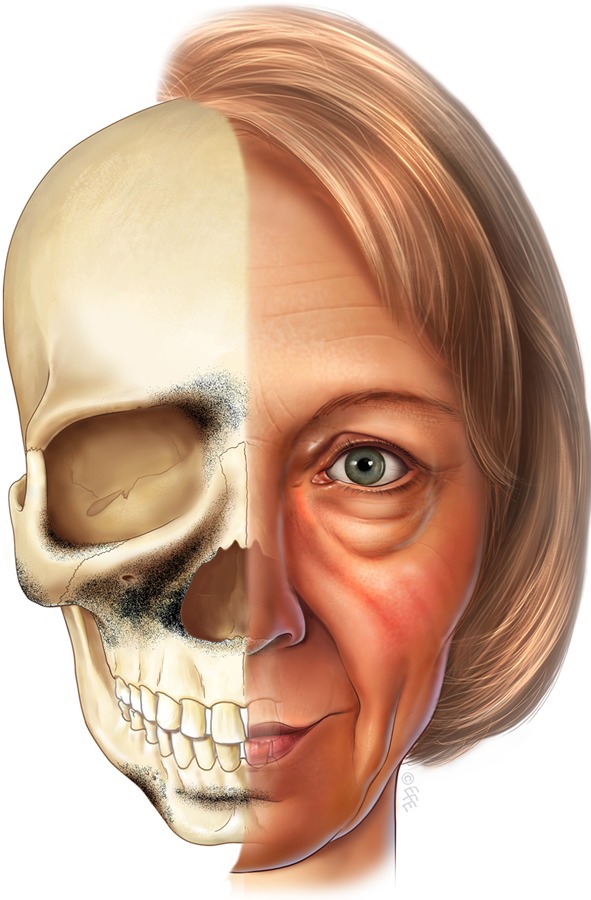
The *darker areas* are those of the greatest bone loss. The stigmata of aging, manifested by the facial soft tissues, corresponds with the areas of weakened skeletal support

The areas most affected by reduced skeletal prominence correspond to those areas of the face that manifest the most prominent stigmata of aging [36] (Fig. 6). In the medial aspect of the upper lid, the brow position is noted to ascend paradoxically with aging, exaggerating the drooped look of the lateral brow [37]. The medial orbital fat pad also becomes more prominent with age, possibly associated with the recession of the superomedial orbital rim. The midcheek manifests the most complex soft tissue changes with aging. The development of the tear-trough deformity, malar mounds, and prominent nasolabial fold and groove may to a significant degree be attributed to the loss of projection of the maxilla with aging [12, 30]. The changes over the lower face are less complex, with the jowl appearing more prominent in relation to the area of reduced skeletal support in the prejowl area of the mandible [29].

In the midcheek, weakness of skeletal support medially contributes to the “tear-trough deformity” [24], and over the malar segment laterally, the changes manifest as malar mounds [38]. Additionally, the major loss of bone projection of the maxilla that contributes to the edge of the pyriform aperture, particularly inferiorly, results in less support of the alar base and upper lip part of the nasolabial groove. Whereas the traditional focus of nasolabial correction has always been on the descent of the heavy fold and the redraping of this from above, it may be more logical to correct the depth of the upper lip position secondary to skeletal changes.

The question of why certain sites are more prone to bony resorption than others has been the subject of little discussion. The maxilla is the bone that undergoes the most dramatic resorption with aging, the consequences of which are seen in the aging mid cheek. The maxilla differs in origin and function from the other bones that make up the orbital rim, being a bone of dental origin. In youth, the maxilla expands to accommodate the growing secondary dentition, which develops within the bone. Eruption of the secondary dentition results in a major reduction of the maxilla’s volume, especially in its lower part.

It is reasonable to speculate that the reverse, i.e., a lack of stress, may be a factor contributing to bone losses in these areas [4, 5]. It is interesting then to note that the sites identified as areas prone to bony resorption in the facial skeleton correspond to the more mobile part of the face during animation, especially the obicularis oculi covering the lateral brow, the lateral orbital crow’s feet areas, and the inferolateral orbital rim. The mobility required for the function of these regions is structurally associated with a less ligamentous fixation of the soft tissues to the bone. Hence, the attachment of the muscles and ligament to the bone in these areas is attended by little stress. It is reasonable to speculate that the opposite, a lack of stress, may be a factor contributing to bone losses in these areas.

Some people inherently “age better” than others. These individuals can be recognized in youth because they have a more attractive face with a strong skeletal structure, as evidenced by the presence of youthful bony features that provide good support to the overlying soft tissues. These features include a prominent supraorbital bar, a strong cheekbone, and a prominent jaw line [36, 38, 39]. Because these individuals innately have youthful bony contours, they start off “high on the curve” of bony support so that it takes longer for the bone loss of aging to manifest clinically.

Conversely, people with poor facial skeletal support never have the ideal contours of youth and start off lower down the curve, and many appear old for their age, even in their 20’s. They are effectively predisposed to manifest aging changes prematurely.

Features that portend poor support include a retrusive supraorbital bar, midface hypoplasia, poor zygomatic development (the extreme seen in Treacher Collins syndrome), and microgenia. Regardless of age, once these soft tissue changes are manifest, a significant degree of bony deficiency exists. Correction of the bony element has the potential to deliver a more harmonious facial rejuvenation.

Those individuals who started low down the curve should be considered for a skeletal augmentation, not only to reverse the aging changes but also to bring their natural state higher up the curve so that the soft tissues are better supported for a more attractive look. With more youth, these individuals have more in reserve against future aging. Facial bone structures also explain the observation that we tend to age like our parents, a familial trait that we inherit from them.

In recent years, augmentation for selected areas of the facial skeleton has become a powerful adjunct in our approach to facial rejuvenation. Although many materials can be used, we generally prefer the use of porous hydroxyapatite granules (Interpore International, Irvine, CA, USA) for their versatility and due to proven clinical experience. Hydroxyapatite is biocompatible, having the same mineral composition as bone, and in its porous form, hydroxyapatite is not prone to resorption because it supports fibrovascular ingrowth [40–45], contributing to long-term stability [46]. The granular form provides surgical flexibility, enabling small volume enhancements if need be, yet with minimal subperiosteal dissection. We have found this approach of directly addressing the underlying deficiency to be preferable to techniques that attempt to camouflage these changes such as extensive tissue redraping alone or the use of supraperiosteal soft tissue fillers.

Certain bony features are subjectively perceived as masculine or feminine. In surgical enhancement of the skeleton, it is important to recognize this as a foremost consideration in rejuvenative procedures for either gender. Exaggeration of masculine features is not necessarily attractive in males. Perrett et al. [47] noted that a more feminized face on a male was deemed more attractive than a more masculinized face. This was true for both male and female observers and across different cultures. Moreover, these findings have been confirmed by others [15, 48]. The implication of this is that for both male and female patients, augmentation of the facial skeleton should be done conservatively, with just sufficient volume to restore the contours of youth. Exaggerated augmentations should be avoided.

A significant limitation with use of the currently available data should be recognized. The ideal study would be linear to show the aging changes in the same individuals. With few exceptions, the available studies in this area are cross-sectional population studies based on comparisons among groups of individuals across different ages. This has inherent shortcomings due to the wide variation between normal individuals. A definitive documentation using longitudinal studies hopefully will be available in the future. Also, it is difficult to quantify the complex three-dimensional changes of the aging bone when two-dimensional measurements are used, as is the case in most of the published studies.

## Conclusions

The facial skeleton has a profound effect on an individual’s appearance. A defining characteristic of youth is good skeletal structural support. Facial aging results from a combination of soft tissue and bony changes, with bone loss in specific areas of the facial skeleton contributing significantly to the features of the aging face. This comprehensive review highlights the specific areas known to resorb with aging. It is conceptually important to appreciate that in most individuals with premature aging, the facial skeleton can be inherently inadequate. Accordingly, the changes in the facial skeleton that result from the aging process must be addressed to obtain a natural-appearing facial rejuvenation.

## Electronic supplementary material

Below is the link to the electronic supplementary material.

Supplementary material 1 (MP4 2113 kb)
